# Reverse-sequence endoscopic nipple-sparing mastectomy with immediate implant-based breast reconstruction: an improvement of conventional minimal access breast surgery

**DOI:** 10.3389/fonc.2024.1366877

**Published:** 2024-03-06

**Authors:** Kawun Chung, Yanyan Xie, Faqing Liang, Mengxue Qiu, Huanzuo Yang, Qing Zhang, Hui Dai, Zhenggui Du

**Affiliations:** ^1^ Department of General Surgery, West China Hospital, Sichuan University, Chengdu, China; ^2^ Breast Center, West China Hospital, Sichuan University, Chengdu, China

**Keywords:** C-E-NSM, R-E-NSM, operation time, complication, HUAXI hole 1

## Abstract

**Background:**

Our center proposes a new technique that effectively provides space to broaden the surgical field of view and overcomes the limitations of endoscopy-assisted nipple-sparing mastectomy (E-NSM) by changing the dissection sequence and combining it with air inflation. The purpose of this study was to compare the clinical outcomes of the new technique designated “reverse-sequence endoscopic nipple-sparing mastectomy (R-E-NSM) with subpectoral breast reconstruction (SBR)“ and the conventional E-NSM (C-E-NSM) with SBR.

**Method:**

All patients undergoing E-NSM with SBR at our breast center between April 2017 and December 2022 were included in this study. The cohort was divided into the C-E-NSM group and the R-E-NSM group. The operation time, anesthesia time, medical cost, complications, cosmetic outcomes, and oncological safety were compared.

**Results:**

Twenty-six and seventy-nine consecutive patients were included in the C-E-NSM and R-E-NSM groups, with average ages of 36.9 ± 7.0 years and 39.7 ± 8.4 years (P=0.128). Patients in the R-E-NSM group had significantly shorter operation time (204.6 ± 59.2 vs. 318.9 ± 75.5 minutes, p<0.001) and anesthesia time (279.4 ± 83.9 vs. 408.9 ± 87.4 minutes, p<0.001) and decreased medical costs [5063.4 (4439.6-6532.3) vs. 6404.2 (5152.5-7981.5), USD, p=0.001] and increase SCAR-Q scores (77.2 ± 17.1 vs. 68.8 ± 8.7, P=0.002) compared to the C-E-NSM group. Although trends increased in both the excellent rate of Ueda scores (53.8% vs. 42.3%, P = 0.144), excellent rate of Harris scores (44.0% vs. 63.1%, P=0.102), and decreased surgical complications (7.6% vs. 19.2%, P = 0.135) were observed in the R-E-NSM group, the differences were not significant. There were no significant differences in oncological outcomes between the two groups.

**Conclusion:**

R-E-NSM improves cosmetic outcomes and efficiency of C-E-NSM, reduces medical costs, and has a trend of lower surgical complications while maintaining the safety of oncology. It is a safe and feasible option for oncological procedures that deserves to be promoted and widely adopted in practice.

## 1 Introduction

Endoscopy-assisted nipple-sparing mastectomy (E-NSM) is a minimal access breast surgery that minimizes and hides the scar inconspicuously, thus optimizing aesthetic outcomes and patient satisfaction (1, 2). Moreover, E-NSM has also demonstrated acceptable oncological safety in some studies (3). However, E-NSM has been controversial as a treatment option, mainly because popular working space-building methods such as liposuction and/or skin lifting systems have some drawbacks (4–6). In addition, E-NSM often requires additional instruments and increases interference, operation time, surgical trauma, and medical costs (6, 7). Furthermore, the superficial nature of the breast and the relatively low morbidity of conventional open breast surgery could also be factors (8).

Surgeons at our center have also tried to conduct E-NSM using retractors to raise the pectoralis major muscle, gland, and skin. However, as in some reports, complications are increased due to uneven stress and unclear dissection layers, and the operation time is very long (9, 10). Through the sum of our experience, our team proposed an innovative technique that involves a changed dissection sequence and incorporates air-insufflation for submuscular space and gland dissection; the technique is named “reverse-sequence endoscopic nipple-sparing mastectomy (R-E-NSM).” Our previous studies have demonstrated that R-E-NSM could create sufficient space, improve dissection access, and avoid interference of instruments. For some challenging part dissection areas, such as the inner-lower quadrant, a small accessory incision (HUAXI hole 1) located at the upper-outer quadrant margin of the areola was introduced so that the final operation time was significantly shortened (11, 12). Because no particular instrument is needed and the operation time is short, this technology is widely accepted in China (13), and we even perform R-E-NSM with breast reconstruction surgeries in the 24-hour surgery center (14–17). We have also used this technology to perform breast reconstruction with endoscopic harvesting of the latissimus dorsi flap (18, 19). Up to now, we have considered R-E-NSM to address the drawbacks of conventional E-NSM (C-E-NSM) and expect it to become a standard surgical procedure. To demonstrate the superiority of the R-E-NSM technique, in this retrospective analysis study, we compared the efficiency, oncological safety, surgical safety, and cosmetic results of patients who underwent C-E-NSM with those of patients who underwent R-E-NSM, followed in each case by subpectoral breast reconstruction (SBR).

## 2 Method

### 2.1 Patients

All patients who underwent C-E-NSM and R-E-NSM followed by SBR between April 2017 and December 2022 at the Breast Center, West China Hospital, Sichuan University, by a single physician were consecutively included in this study. A research assistant prospectively collected and recorded all data for the E-NSM patient database, which was then verified by the principal investigator. This study collected data regarding demographic, clinicopathologic, surgical, and therapeutic characteristics, hospitalization time, medical cost, postoperative complications, aesthetic outcomes, recurrence, metastasis, and survival status. The study was approved by the Biomedical Ethics Committee of West China Hospital, Sichuan University (No. 2022-570). An exemption from the requirement for informed consent was granted due to the use of retrospective data. Photos and videos of several patients who consented to the release of their photographs were included in the current research.

The inclusion criteria were as follows: breast cancer in early stages, with a breast tumor less than 5 cm, cN0-1, no skin or chest infiltration, and no evidence of distant metastasis on clinical or radiographic examination. A prophylactic mastectomy was performed in women with RCA1/2 mutations, high-risk lesions such as lobular carcinoma *in situ*, atypical ductal hyperplasia, or women who desired a risk-reducing mastectomy after risk counseling. When the tumor involvement of the nipple-areola complex (NAC) was suspected by intraoperative sub-nipple biopsy, skin-sparing mastectomy was performed rather than NSM.

**Figure 1:**
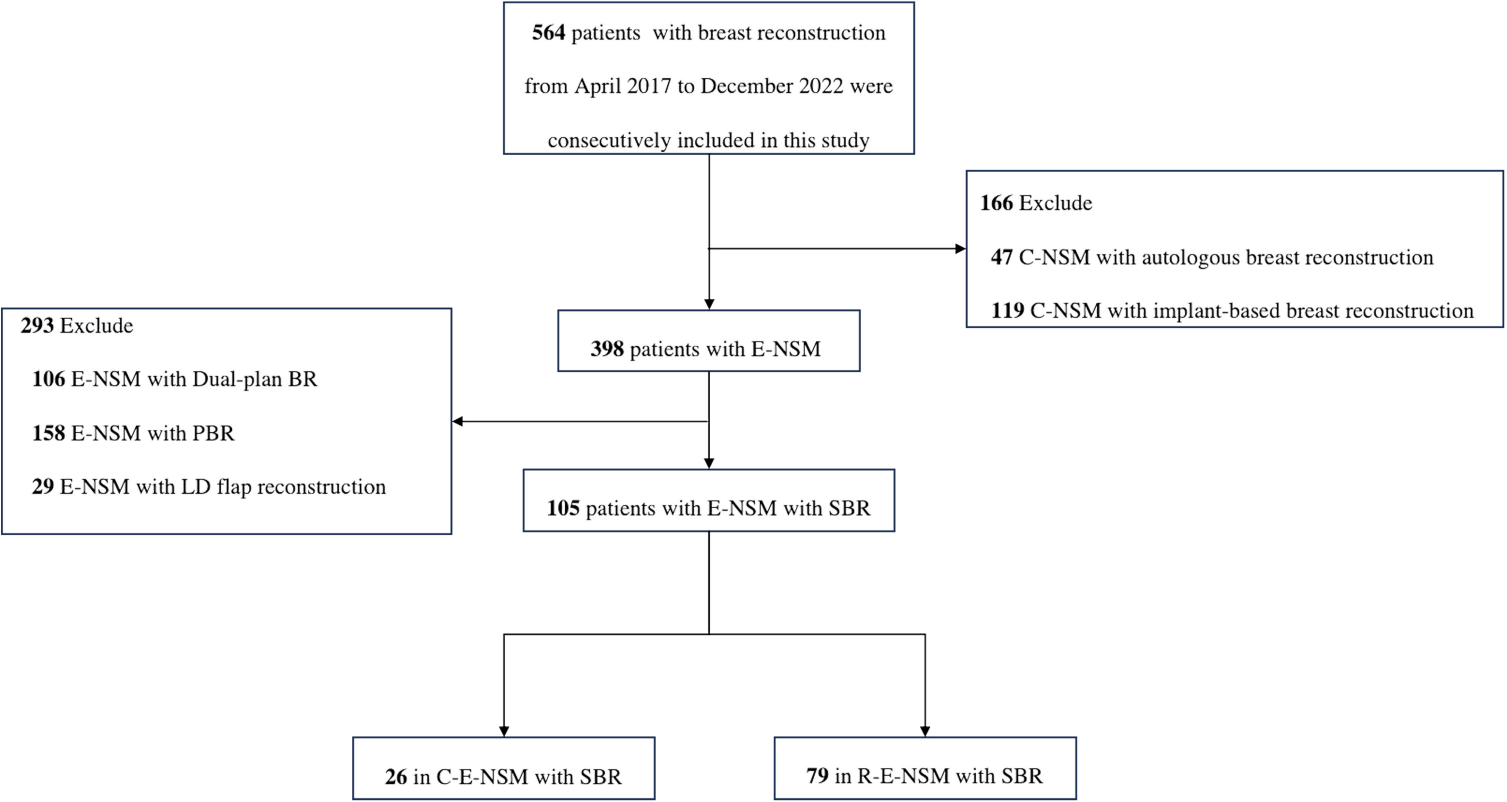
Flow chart of participant selection. C-NSM, conventional nipple-sparing mastectomy; E-NSM, endoscopy-assisted nipple-sparing mastectomy; Dual-plan BR, subpectoral dual-plane implant-based breast reconstruction; PBR, prepectoral implant-based breast reconstruction; LD, latissimus dorsi; SBR, subpectoral implant-based breast reconstruction; C-E-NSM, conventional endoscopy-assisted nipple-sparing mastectomy; R-E-NSM, reverse-sequence endoscopic nipple-sparing mastectomy.

Surgery was contraindicated in patients with severe comorbidities (such as organ dysfunction and immune deficiency) and a poor general condition. We also recommend this procedure for patients with breasts small to moderate in size and mild or no ptosis because unilateral SBR is not recommended for patients with large breasts or moderate-severe ptosis. Because no patients underwent dual-plane implant-based breast reconstruction (dual-plane BR) or prepectoral implant-based breast reconstruction (PBR) in the C-E-NSM group, patients treated with dual-plane BR and PBR were also excluded for the reason that different reconstruction techniques could affect the operation time. The flow chart of participant selection is shown in **Figure 1**.

### 2.2 C-E-NSM operation method

**Figure 2:**
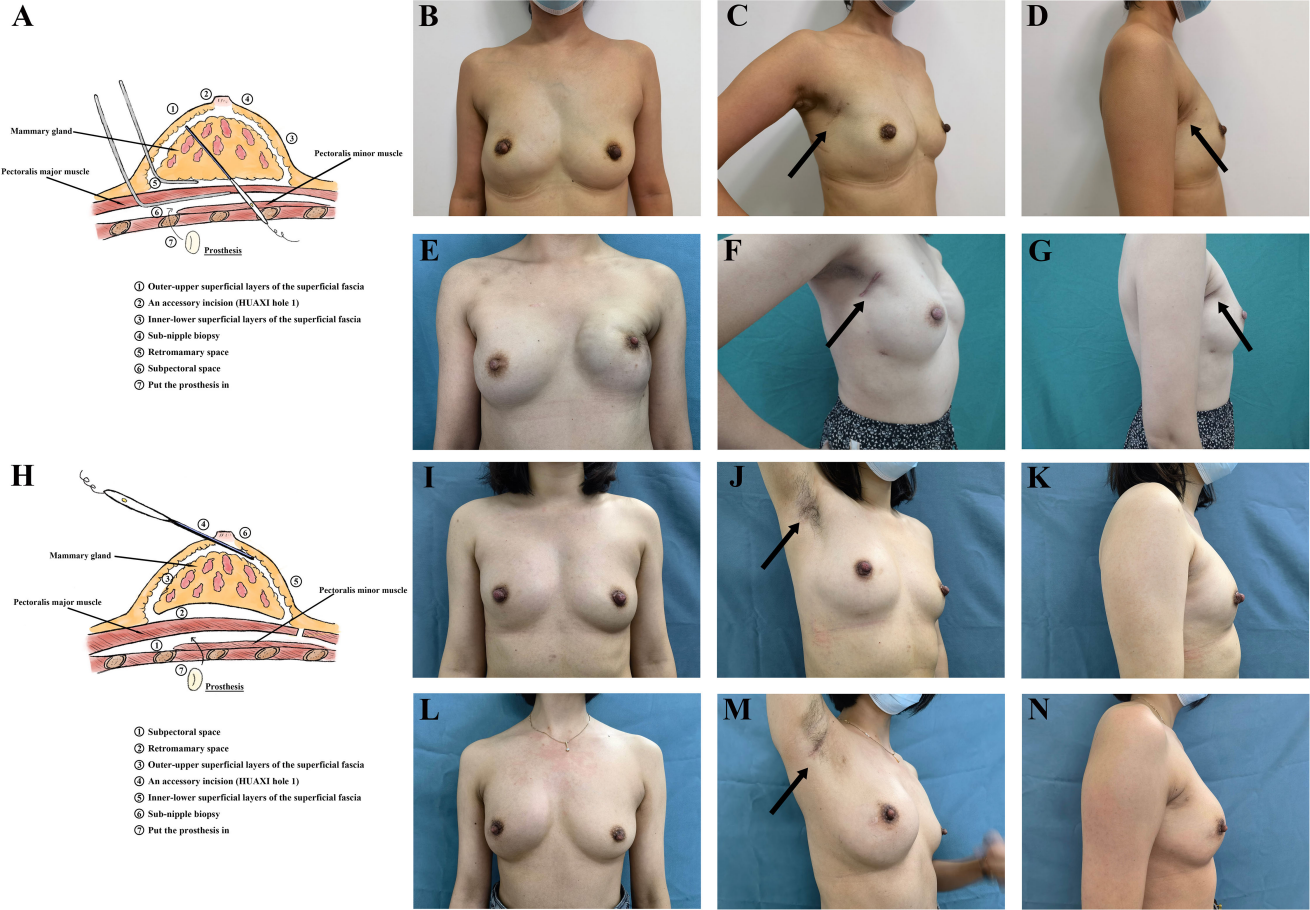
**(A)** The process of the C-E-NSM with SBR. **(B–D)** Photographs of a patient with right breast cancer who underwent C-E-NSM with SBR three years ago. **(E–G)** Photographs of a patient who received bilateral C-E-NSM with SBRs 2.5 years ago. The incision is located on the outer-upper edge of the breast, and the scar can be visible when patients stand erect and hang their arms naturally. **(H)** The process of the R-E-NSM with SBR. **(I–N)** Photographs of two patients with right breast cancers who received the C-E-NSM with SBRs 2.5 years and 2 years ago, respectively. The incision is located on the top of the axilla’s natural fold, and no scar is visible when patients stand erect and hang their arms naturally. C-E-NSM, conventional endoscopy-assisted nipple-sparing mastectomy; R-E-NSM, reverse-sequence endoscopic nipple-sparing mastectomy. SBR, subpectoral implant-based breast reconstruction.

Incisions are made along the outer upper edge of the breast (**Figures 2A–G**). Under direct vision, axillary lymph node surgery is performed. The gland dissection sequence is just the same as conventional open surgery, which begins with the superficial layer of superficial fascia, followed by the pectoralis major fascia. These two layers should be dissected under direct vision as far as possible, and the endoscope should be replaced if direct vision is unclear. When the gland is removed, an intraoperative sub-nipple biopsy is taken, and the pectoralis major muscle is lifted with a retractor to build the implant envelope in the post-pectoralis major muscle space. The prosthesis is placed after flushing all layers, and the operation is complete after inserting the indwelling drainage tube and closing the incision.

### 2.3 R-E-NSM operation method

An incision is made at the top of the axilla’s natural fold (**Figures 2H–N**). Dissection of the axillary lymph nodes or sentinel lymph node biopsy (SLNB) is performed under direct vision. In contrast to C-E-NSM, the dissection sequence is reversed, and total endoscopic surgery is utilized. The dissection sequence begins from the subpectoral plane to build the implant envelope. By setting the air pressure to 12 mmHg and the flow rate to 40 L/mL, carbon dioxide (CO_2_) elevates the pectoralis major muscle and the entire breast. The subpectoral space is dissected using an electric scalpel from the parasternal line to 1cm beyond the inframammary fold marked inferiorly and to the serratus anterior fascia laterally. Then, an electric scalpel is used to dissect the entire pectoralis major fascia with the help of a grasper to pull the pectoralis major muscle downward. Next, skin flap dissection is performed, and an electric scalpel is used to carefully dissect the outer-upper, inner-upper, and outer-lower superficial layers of the superficial fascia. A 2 mm incision is made in the upper-outer margin of the areola (HUAXI hole 1), which makes it possible to remove the inner-lower part of the gland completely. Through the axillary incision, the entire gland is excised and removed. Then, a biopsy is taken from under the NAC with direct visibility for frozen sections. The prosthesis is placed after flushing all layers, and the operation is completed after the indwelling drainage tube is inserted. Full details are given in **Video 1**. There are some videos from different views of the camera in some articles we have published (16, 17, 20).

### 2.4 Outcome measures

The following outcome indicators were compared between the two groups: operation time (starting when the physician makes the first incision and ending when sutures are finished), anesthesia time, and medical cost. Surgical safety mainly included complications, which were considered “minor” if they were of Clavien-Dindo classification (C-D-C) grades I or II, and “major” if they were of C-D-C grades III or IV. The oncological safety included local recurrence and distant metastasis at the last follow-up. The patient-reported cosmetic outcomes include the Harris and SCAR-Q scores (19, 21). The appearance scale from the SCAR-Q self-report questionnaire comprises 12 questions to appraise patient satisfaction with a surgical scar when complete healing had taken place; only operative side scars were scored. Higher scores indicate a better cosmetic outcome. Regarding the doctor-reported cosmetic outcomes, three surgeons who did not participate in the surgery adopted the Ueda scoring system to evaluate preoperative and postoperative photographs (22).

### 2.5 Statistical analysis

The continuous variables were analyzed using the independent t-test and calculated as the mean ± standard deviation. Skewed distribution data are shown as the median and were compared with a nonparametric test. The difference in categorical variables was compared using the Chi-square or Fisher’s exact test analysis of variance. The Wilcoxon rank sum test was used for orderly classified data. Analysis of risk factors for outcome indicators by univariate and multivariate analyses and logistic regression was used to estimate ORs and 95% CIs. Statistical tests were 2-sided, and a P value less than 0.05 was considered statistically significant. All measurements and calculations were analyzed using SPSS 25.0.

## 3 Results

### 3.1 Patient demographics and characteristics

A total of 105 patients who underwent E-NSM with SBR in our breast center between April 2017 and December 2022 were consecutively included in this study. There were 26 patients whose average age was 36.9 ± 7.0 years in the C-E-NSM group and 79 patients whose average age was 39.7 ± 8.4 years in the R-E-NSM group (p=0.128). The comparison between the C-E-NSM group and the R-E-NSM group showed a statistically significant difference in BMI (p=0.016), whether to use an accessory incision (HUAXI hole 1) (p<0.001) and breast ptosis (p<0.007). More patients in the C-E-NSM group than in the R-E-NSM group received neo-adjuvant chemotherapy (p=0.006), adjuvant chemotherapy (p=0.047), and adjuvant radiotherapy (p=0.017). The median follow-up time was 35 months (range 30-63) in the C-E-NSM group and 25 months (range 12-31, p<0.001) in the R-E-NSM group, as described in **Table 1**.

**Table 1:** Clinical and demographic characteristics of 105 patients in the C-E-NSM and R-E-NSM.

Variable	C-E-NSM (n=26)	R-E-NSM (n=79)	P
Age (x ± sd, year)	36.9 ± 7.0	39.7 ± 8.4	0.128
BMI (x ± sd, kg/m^2^)	22.0 ± 2.6	20.6 ± 2.5	**0.016**
Implant size (median, range, cc)	280 (140-425)	245 (125-460)	0.174
Accessory incision (HUAXI hole 1)			**<0.001**
Yes	10 (38.5)	76 (96.2)	
No	16 (61.5)	3 (3.8)	
Location			0.687
Unilateral	17 (65.4)	55 (69.6)	
Bilateral	9 (34.6)	24 (30.4)	
Breast/Ovarian cancer family history			1.000
Yes	2 (7.7)	7 (8.9)	
No	24 (92.3)	72 (91.1)	
Menopause			0.686
Yes	3(11.5)	6(7.6)	
No	23(88.5)	73(92.4)	
Cup size			0.196
≤A	5 (19.2)	29 (36.7)	
B	16 (61.5)	34 (43.0)	
C-D	5 (19.2)	16 (20.3)	
Ptotic breast			**0.007**
Yes	1 (3.8)	23 (29.1)	
No	25 (96.2)	56 (70.9)	
Pathologic T stage (NA=4) ^a*^			0.312
Tis and T1	21 (80.8)	48 (69.6)	
T2	5 (19.2)	17 (21.5)	
Pathologic N stage ^a^			0.168
N0	16 (61.5)	60 (79.7)	
N1-3	10 (38.5)	16 (20.3)	
ER (NA=3) ^a^			0.124
Positive	17 (65.4)	57 (82.6)	
Negative	8 (30.8)	10 (14.5)	
PR (NA=3) ^a^			0.436
Positive	18 (69.2)	55 (80.9)	
Negative	7 (26.9)	11 (16.2)	
HER2 (NA=2) ^b^			0.585
Positive^@^	4 (15.4)	14 (20.6)	
Negative	21 (80.8)	53 (77.9)	
Ki-67 (NA=7) ^b^			0.827
≤20	13 (50.0)	33 (48.5)	
>20	12 (46.2)	29 (42.6)	
Subtypes (NA=3) ^b^			0.138
Lumina A	9 (34.6)	33 (48.5)	
Lumina B	8 (30.8)	18 (26.5)	
Her2	4 (15.4)	11 (16.2)	
TNBC	4 (15.4)	4 (5.9)	
Axillary lymph node management			0.130
Untreated	0	7 (8.9)	
SLNB	18 (69.2)	57 (72.2)	
ALND	8 (30.8)	15 (19.0)	
Nipple resection			0.732
Yes	4 (15.4)	9 (11.4)	
No	22 (84.6)	70 (88.6)	
Nipple reconstruction			1.000
Yes	2 (7.7)	7 (8.9)	
No	24 (92.3)	72 (91.1)	
Neo-adjuvant chemotherapy	8 (30.8)	6 (7.6)	**0.006**
Adjuvant chemotherapy	16 (61.5)	31 (39.2)	**0.047**
Adjuvant radiotherapy	11 (42.3)	15 (19.0)	**0.017**
Adjuvant endocrinotherapy	16 (61.5)	58 (73.4)	0.249
Anti-Her2 therapy	4 (15.4)	12 (15.2)	1.000
Follow-up time (median IQR, month)	35 (30-63)	25 (12-31)	**<0.001**

C-E-NSM, conventional endoscopy-assisted nipple-sparing mastectomy; R-E-NSM, reverse-sequence endoscopic nipple-sparing mastectomy; SD, standard deviation; BMI, body mass index; IQR, interquartile range; ER, oestrogen receptor; PR, progesterone receptor; HER-2, human epidermal growth factor receptor 2; TNBC, triple-negative breast cancer; SLNB, sentinel lymph node biopsy; ALND, axillary lymph node dissection.

*, 7 cases of Tx stage.

^@^, including +++, ++, and FISH amplification.

^a^, only including malignant cases.

^b^, only including invasive breast cancer.

Bold values indicate p<0.05.

### 3.2 Perioperative parameters and medical cost

**Figure 3:**
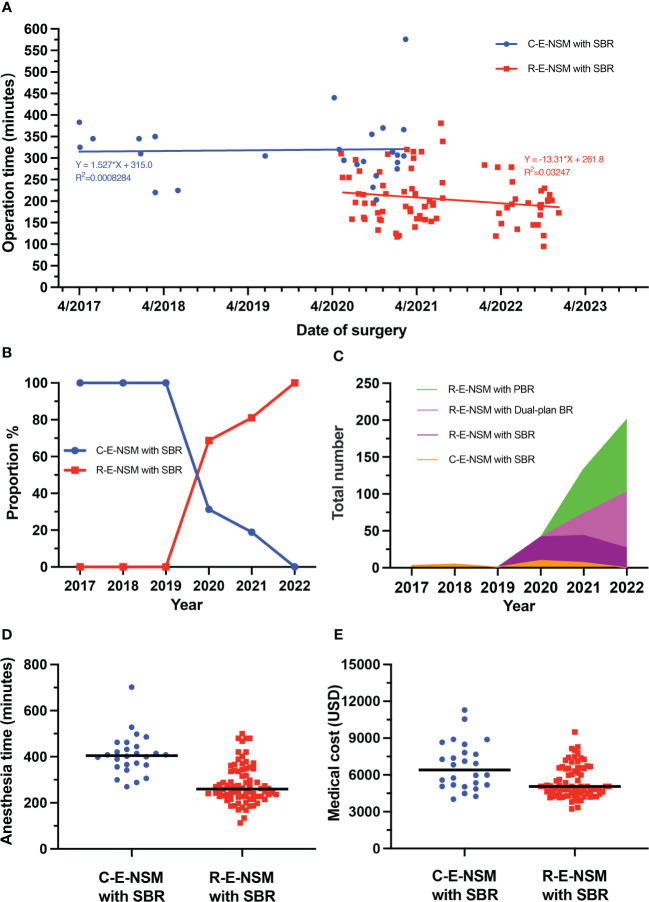
**(A)** The operation times of patients undergoing C-E-NSM and R-E-NSM with PBR, respectively, between April 2017 and December 2022. **(B)** The proportion between C-E-NSM and R-E-NSM from 2017 to 2022 in our medical team. **(C)** The total number of endoscopic nipple-sparing mastectomy followed by implant-based breast reconstruction. **(D)** The anesthesia times of patients undergoing C-E-NSM and R-E-NSM. **(E)** The medical cost of patients undergoing C-E-NSM and R-E-NSM. C-E-NSM, conventional endoscopy-assisted nipple-sparing mastectomy; R-E-NSM, reverse-sequence endoscopic nipple-sparing mastectomy; SBR, subpectoral implant-based breast reconstruction; Dual-plan BR, subpectoral dual-plane implant-based breast reconstruction; PBR, prepectoral implant-based breast reconstruction.

A comparison of the R-E-NSM group and the C-E-NSM group showed that the operation time was reduced significantly (204.6 ± 59.2 vs. 318.9 ± 75.5 minutes, p<0.001) (**Figure 3A**). As a result of the shortened operation time and improved efficiency, the ratio of R-E-NSM with SBR increased, and the ratio of C-E-NSM with SBR decreased in our medical team (**Figure 3B**). The total number of R-E-NSM also increased significantly, despite the number of R-E-NSM with SBR procedures slightly decreased during 2021-2022 because of the great promotion of R-E-NSM with subpectoral dual-plane BR or PBR by changing surgical concepts (**Figure 3C**). The anesthesia time (279.4 ± 83.9 vs. 408.9 ± 87.4 minutes, p<0.001) (**Figure 3D**) and medical cost [5063.4(4439.6-6532.3) vs. 6404.2(5152.5-7981.5), USD, p=0.001] (**Figure 3E**) were also significantly reduced in the R-E-NSM group compared to C-E-NSM group.

To adjust the bias of baseline and explore independent risk factors affecting operation time, the cohort of patients was divided into two groups based on average operation time (232 minutes) for univariate and multivariate logistic regression analysis. Factors (P < 0.1) significant on univariate analyses were subjected to multivariate logistic regression analysis, including dissection sequence, follow-up time, BMI, implant size, accessory incision (HUAXI hole 1), and unilateral or bilateral operation. The independent risk factors for the operation time are illustrated in **Table 2**, which were dissection sequence (OR, 0.110; 95% CI, 0.021-0.588, p=0.010) and location (OR, 13.73; 95% CI, 3.891-48.463, P <0.001).

**Table 2:** Univariate and multivariate analysis of the parameters resulting in operation times longer than the average time (232 minutes).

Variable	Univariate analysis			Multivariate analysis		
	OR	95% Cl	P	OR	95% Cl	P
Dissection sequence			**<0.001**	0.110	0.021-0.588	**0.010**
C-E-NSM	Ref					
R-E-NSM	0.062	0.019-0.201				
Age (x ± sd, y)	0.972	0.925-1.022	0.650			
Follow-up time (median IQR, month)	1.072	1.031-1.115	**<0.001**	1.049	0.987-1.116	0.124
BMI (x ± sd, kg/m2)	1.1800	1.005-1.384	**0.043**	1.085	0.833-1.415	0.545
Implant size	1.005	0.999-1.011	**0.097**	1.000	0.991-1.010	0.979
Accessory incision (HUAXI hole 1)			**<0.001**			0.480
Yes	Ref					
No	20.740	4.458-96.484		0.504		
Location			**<0.001**			**<0.001**
Unilateral	Ref					
Bilateral	6.416	2.586-15.919		13.731	3.891-48.463	
Breast/Ovarian cancer family history			0.776			
Yes	Ref					
No	0.819	0.207-3.245				
Menopause			0.326			
Yes	Ref					
No	0.502	0.127-1.989				
Cup			0.665			
≤A	Ref					
B	1.222	0.496-3.014				
C-D	1.667	0.550-5.048				
Degree of ptosis			0.449			
Yes	Ref					
No	1.447	0.556-3.766				
T stage ^a*^			0.119			
Tis and T1	Ref					
T2	0.434	0.151-1.240				
N stage			0.402			
N0	Ref					
N1≥	1.469	0.597-3.618				
ER ^a^			0.300			
Positive	0.542	0.191-1.532				
Negative	Ref					
PR ^a^			0.158			
Positive	0.417	0.146-1.188				
Negative	Ref					
HER2^b^			0.524			
Overexpression	0.776	0.262-2.297				
Negative	Ref					
Ki-67^b^			0.553			
≤20	Ref					
>20	1.467	0.618-3.486				
Subtypes ^b^			0.248			
Lumina A	Ref					
Lumina B	1.250	0.452-3.459				
Her2	1.000	0.286-3.492				
TNBC	6.000	1.070-33.645				
Axillary lymph node management			0.394			
Untreated	Ref					
SLNB	1.510	0.275-8.296				
ALND	2.750	0.432-17.489				
Nipple mastectomy			0.282			
Yes	Ref					
No	0.526	0.164-1.694				
Nipple reconstruction			0.326			
Yes	Ref					
No	0.502	0.127-1.989				
Neo-adjuvant chemotherapy	2.235	0.715-6.993	0.167			

C-E-NSM, conventional endoscopy-assisted nipple-sparing mastectomy; R-E-NSM, reverse-sequence endoscopic nipple-sparing mastectomy; SD, standard deviation; BMI, body mass index; IQR, interquartile range; OR, odds ratio; ER, estrogen receptor; PR, progesterone receptor; HER-2, human epidermal growth factor receptor 2; TNBC, triple-negative breast cancer; SLNB, sentinel lymph node biopsy; ALND, axillary lymph node dissection; Ref, reference.

*, 7 cases of Tx stage.

^a^, only including malignant cases.

^b^, only including invasive breast cancer. Bold values indicate p<0.05.

### 3.3 Complications and oncological outcomes

In comparing the C-E-NSM and R-E-NSM groups, there were no significant differences in terms of surgical complications (19.2% vs. 7.6%, p=0.135), major complications (7.7% vs. 1.3%, p=0.151), minor complications (19.2% vs. 7.6%, p=0.135), and implant-related complications (23.1% vs. 20.3%, p=0.759) (**Table 3**). There were no implant loss or flap necrosis cases in the R-E-NSM group. According to univariate analyses of any complications, two factors showed a p <0.1, and multivariate analyses indicated that the follow-up time was the only independent risk factor for any complication (OR,1.015; 95% CI, 1.015-1.095; P = 0.007) (**Table 4**). There was one local recurrence and two distant metastasis cases in the C-E-NSM group. In contrast, there were no local recurrence or distant metastasis cases in the R-E-NSM group. A two-year disease-free survival (DFS) rate of 96.2% and 100% was observed in the C-E-NSM and R-E-NSM groups, respectively (P=0.248).

**Table 3:** Comparison of the complications and aesthetic outcomes in C-E-NSM and R-E-NSM.

Variable	C-E-NSM (n=26)	R-E-NSM (n=79)	P
Any surgical complication	5 (19.2)	6 (7.6)	0.135
Major complications	2 (7.7)	1 (1.3)	0.151
SSI	1 (3.8)	1 (1.3)	
Implant loss	1 (3.8)	0	
flap necrosis	1 (3.8)	0	
Minor complications	5 (19.2)	6 (7.6)	0.135
Seroma	3 (11.5)	1 (1.3)	
SSI	1 (3.8)	4 (5.1)	
Hemorrhage	1 (3.8)	2 (2.5)	
NAC necrosis	1 (3.8)	1 (1.3)	
Implant complications	6 (23.1)	16 (20.3)	0.759
Deformity of movement	0	1 (1.3)	1.000
Ectopectoralis spasm	2 (7.7)	3 (3.8)	0.595
Ectopectoralis pain	2 (7.7)	10 (12.7)	0.726
Rippling	0	3 (3.8)	0.573
Prosthesis outline appear	3 (11.5)	5 (6.3)	0.406
Capsular contracture^*^			0.105
1	1 (3.8)	4 (5.1)	
2	5 (19.2)	7 (8.9)	
3	5 (19.2)	6 (7.6)	
Harris score (NA=15)			0.102
Excellent	11 (44.0)	41 (63.1)	
Good	9 (36.0)	16 (24.6)	
General	4 (16.0)	7 (10.8)	
Poor	1 (4.0)	1 (1.5)	
Ueda score (NA=14)			0.144
Excellent	11 (42.3)	35 (53.8)	
Good	10 (38.5)	27 (41.5)	
General	4 (15.4)	2 (3.1)	
Poor	1 (3.8)	1 (1.5)	
SCAR-Q (NA=9)	68.8 ± 8.7	77.2 ± 17.1	**0.002**

C-E-NSM, conventional endoscopy-assisted nipple-sparing mastectomy; R-E-NSM, reverse-sequence endoscopic nipple-sparing mastectomy; SD, standard deviation; IQR, interquartile range; min, minute; SSI, surgery site infection; NAC, nipple-areola complex;.

*: reference Baker grading system. Bold value indicates p<0.05.

**Table 4:** Univariate and multivariate analysis of any surgical complication.

Variable	Univariate analysis			Multivariate analysis		
	OR	95% Cl	P	OR	95% Cl	P
Follow-up time (median IQR)	1.064	1.024-1.105	**0.001**	1.015	1.015-1.095	**0.007**
Adjuvant chemotherapy			**0.019**			0.062
Yes	6.632	1.357-32.409		4.829	0.929-25.216	
No	Ref					

Ref, reference; IQR, interquartile range; min, minute.

univariate analysis outcome p<0.1 shown. Bold values indicate p<0.05.

### 3.4 Aesthetic outcomes

Postoperative aesthetic outcome questionnaires included doctor-reported outcomes (Ueda scale) and patient-reported outcomes (Harris scale and SCAR-Q). Ninety patients completed the questionnaires for the Harris scale, 91 patients completed the questionnaires for the Ueda scale, and 96 patients completed the questionnaires for SCAR-Q. When comparing the C-ENS and R-ENS groups, there was no significant difference in Harris scores (P=0.102) and Ueda scores reported by doctors (P=0.144). However, the mean SCAR-Q score showed a significant difference (68.8 ± 8.7 vs. 77.2 ± 17.1, P=0.002) between the two groups.

## 4 Discussion

This study presents a groundbreaking comparison of two breast cancer surgical procedures, C-E-NSM with SBR and innovation technique R-E-NSM with SBR, in terms of efficiency, safety, and economic results at the first time. The results showed that R-E-NSM outperformed C-E-NSM regarding operation time, anesthesia time, and cost. Moreover, it improved patient satisfaction with the appearance of their scars and revealed a decreasing trend in the incidence of complications. These findings confirm that R-E-NSM is a highly efficient and recommended new surgical procedure that breaks conventional methods. Therefore, R-E-NSM is a promising new surgical procedure that has the potential to revolutionize breast cancer surgery. Given its further development, R-E-NSM is now suitable for a broader range of operations, including subpectoral dual-plane BR, PBR, latissimus dorsi (LD) flap reconstruction, and gynecomastia mastectomy (15, 23, 24).

The operation time was reduced by approximately 2 hours in the R-E-NSM group, and the medical cost and anesthesia time were also reduced compared to C-E-NSM. C-E-NSM increases the difficulty of optimal exposure, such as lifting the pectoralis major muscles, glands, and skin. You cannot have your cake and eat it, too. When the skin-lifting method is chosen, the glands and pectoralis major muscle need to be raised with a retractor and some specific equipment, resulting in poor exposure and difficult dissection of the deep layer. When the air-insufflation method is chosen, using CO_2_ exposure is beneficial, but lifting the pectoralis major muscle and the gland with this method is difficult. Therefore, the process of C-E-NSM with SBR will take longer, approximately 5 hours (10, 25).

In contrast, R-E-NSM can resolve the above issue and reduce the operation time using a reverse dissection sequence and CO_2_ insufflation. Now you can have your cake and eat it too. With CO_2_ insufflation, the field of vision is clearly exposed, and with CO_2_ simultaneously just as a universal retractor, the pectoralis major muscle, gland, and skin are lifted easily. An accessory incision (HUAXI hole 1) can also be combined with R-E-NSM to dissect the inner-lower part of the superficial layers of the superficial fascia and prevent equipment interference. The follow-up time was also the reason for the operation time decrease by univariate analyses. We know that the follow-up time reflects the learning curve. As time passes, the surgeon becomes more proficient and experienced. Currently, we only need 60-90 minutes for R-E-NSM with SBR and approximately 60-75 minutes for R-E-NSM with PBR.

Regarding cosmetic outcomes, the SCAR-Q appearance scale is used to evaluate patient satisfaction after developing a surgical scar. Higher scores indicate a better cosmetic outcome. In the R-E-NSM group, the SCAR-Q score was higher, and it was speculated that the reason was that scarring would be more subtle and concealed under the axillary area. There is an axillary incision with a length of approximately 50 mm, combined with a “HUAXI hole 1”, which has a size of 2 mm. Scarring around the breasts is barely noticeable when patients stand erect and hang their arms naturally. From C-E-NSM (10, 26), the incision was mainly placed on the lateral upper side of the breast. Because of a gasless technique and retractor, the incision cannot be close to the top of the axilla. The incision was extended to 5-8 cm to remove the mammary gland and axillary tissue. As a result, scars cannot be concealed completely. The Harris score and Ueda score were more associated with the appearance of breasts which are reported by patients and doctors, respectively. Because the operation method for all patients was subpectoral implant-based breast reconstruction, there was no significant difference in the appearance of the breasts. However, we can also notice a trend from the result that the cosmetic outcomes with R-E-NSM and SBR were better than those with C-E-NSM and SBR.

This study also showed a downward trend of complications in R-E-NSM. Follow-up time was the only independent factor of any surgical complications after multivariate analysis. Therefore, the decreasing trend of complications is not caused by different surgical methods but due to the shorter follow-up time of R-E-NSM and the improvement of surgical proficiency. However, the current complication rate is 7.6% in the R-E-NSM group; with the progress of surgical skill proficiency, the incidence of complications in the R-E-NSM group may be further reduced. Following E-NSM, flap necrosis and NAC necrosis are common complications. However, in this study, there were reports of no cases of flap necrosis in R-E-NSM, and necrosis of the NAC in the R-E-NSM group was lower than that in the C-E-NSM group (1.3% vs. 3.8%), as reported in other studies and even lower than other studies (27–30). Tissue necrosis is associated with damage to blood vessels and enlarged incisions during the operation, and periareolar incisions were identified as predictors of NAC necrosis (29, 31). However, in the R-E-NSM group, gas can evenly expand tissue and expose it well, reducing the damage to surrounding tissues and blood vessels and combining minimal peri-areola accessory incision (HUAXI hole 1), thereby decreasing necrosis complications. Furthermore, a long operation time is a risk factor for surgery site infection (SSI) (32, 33), so reducing the operation time could reduce the probability of SSI. All of the above are reasons for the trend of decreased complication rate after R-E-NSM compared with C-E-NSM.

Oncological safety is a major concern in the treatment of breast cancer. The endoscopic procedures in this study followed the same steps and principles as conventional nipple-sparing mastectomy (C-NSM). Therefore, we speculate that the oncological results of the two techniques may be similar (34). By a median follow-up time of 35 months, the locoregional recurrence rate was 3.8%, the distant metastasis rate was 7.7%, and no patients in the C-E-NSM group experienced mortality. By a median follow-up time of 25 months, there was no report of local recurrence or distant metastasis from patients in the R-E-NSM group. The two-year DFS rate was 96.2% in the C-E-NSM group and 100% in the R-E-NSM group, which is consistent with several studies of C-NSM (35, 36). However, the rate of local recurrence within two years in the R-E-NSM group was lower than that in the C-E-NSM group, although there was no significant difference. In addition to the small sample size due to accidental factors, it has been suggested that insufficient exposure of the retractor, uneven resection, and residual tumor may contribute to the risk of local recurrence in C-E-NSM. The R-E-NSM dissection is even and ranges wider, making it a potentially safer procedure. These results indicate that the oncologic safety of R-E-NSM is acceptable, though the recurrence and survival outcomes are still pending.

R-E-NSM significantly reduced operation time, anesthesia time, and medical cost and showed a downward trend of complications. In particular, patients and doctors in China widely accept R-E-NSM with subpectoral dual-plane BR and PBR to reduce trauma, shorten surgical time, and expand the indication population. These even make R-E-NSM with implant-based breast reconstruction has been successfully performed in the 24-hour surgical center of our hospital. Nevertheless, as a retrospective study, the investigation involved a limited number of cases and was based on the expertise of a single physician and center, resulting in a bias in patient selection, insufficient representation of the general population, and insufficient samples. It is recommended that further prospective cohort studies or randomized controlled trials be conducted.

## 5 Conclusion

In summary, R-E-NSM improves cosmetic outcomes and efficiency of C-E-NSM, reduces medical costs, and has a trend of lower surgical complications while maintaining the safety of oncology. It is a safe and feasible option for oncological procedures that deserves to be promoted and widely adopted in practice.

## Data availability statement

The original contributions presented in the study are included in the article/**Supplementary Material**, further inquiries can be directed to the corresponding author.

## Ethics statement

The study was approved by the Biomedical Ethics Committee of West China Hospital, Sichuan University (No. 2022-570). An exemption from the requirement for informed consent was granted due to the use of retrospective data.

## Author contributions

KC: Writing – original draft, Data curation, Formal analysis, Methodology, Writing – review & editing. YX: Data curation, Writing – review & editing. FL: Data curation, Formal analysis, Supervision, Writing – review & editing. MQ: Data curation, Writing – review & editing. HY: Data curation, Writing – review & editing. QZ: Data curation, Writing – review & editing. HD: Data curation, Writing – review & editing. ZD: Supervision, Writing – review & editing, Project administration.
